# Management of Acute Severe Colitis in the Era of Biologicals and Small Molecules

**DOI:** 10.3390/jcm8122169

**Published:** 2019-12-08

**Authors:** Christine Verdon, Talat Bessissow, Peter L. Lakatos

**Affiliations:** 1 Division of Gastroenterology, McGill University Health Centre, Montreal, QC H3G 1A4, Canada; christine.verdon@mail.mcgill.ca (C.V.); talat.bessissow@mcgill.ca (T.B.); 2 Department of Gastroenterology, Campbelltown Hospital, Sydney, NSW 2560, Australia; 3 1st Department of Medicine, Semmelweis University, Budapest H1083, Hungary

**Keywords:** acute severe ulcerative colitis, infliximab, cyclosporine, tacrolimus, corticosteroids, surgical management

## Abstract

Acute severe ulcerative colitis (ASUC) is a medical emergency which occurs in about 20%–30% of patients with ulcerative colitis during their lifetime, and does carry a mortality risk of 1%. The management of inflammatory bowel diseases has evolved with changes in objective patient monitoring, as well as the availability of new treatment options with the development of new biological and small molecules; however, data is limited regarding their use in the context of ASUC. This review aims to discuss the emerging data regarding biologicals and small molecules therapies in the context of ASUC.

## 1. Introduction

Acute severe ulcerative colitis (ASUC) is a life-threatening medical emergency which carries a 1% mortality rate [1]. Patients diagnosed with ulcerative colitis (UC) have a lifetime risk of 20%–30% of developing an acute flare of their disease requiring hospitalization [2]. Corticosteroids remain the mainstay of initial therapy but 30%–40% of patients who fail to respond will require second-line salvage treatment with mainly infliximab (IFX) or cyclosporine (CsA) [3,4]. There has been a great evolution in the management of inflammatory bowel diseases (IBD) overall with new molecules becoming available; their use is also being explored in the context of ASUC. Admission under the care of a gastroenterologist has been shown to reduce in-hospital and one-year mortality rates, but not colectomy risk [5]. Colectomy remains a definitive treatment in ulcerative colitis, however colectomy in the context of ASUC carries a higher postoperative mortality risk up to 5.3% [6]. The aim of this review is to discuss the emerging evidence in the management of ASUC.

## 2. Definition, Evaluation and Risk Stratification of ASUC

ASUC is a medical emergency and adequate evaluation of these patients is crucial to ensure prompt treatment. It is therefore of utmost importance to identify these at-risk patients early. Multiple definitions are available for ASUC. Originally, the Truelove and Witts [7] criteria were developed to assess patients on initial admission to hospital. Meanwhile, Travis or Oxford Criteria [8], Ho index [9], Seo index [10] or Swedish score [11] (also called fulminant colitis score) were developed to assess progression at about Day 3 of treatment with first line intravenous (IV) corticosteroids to predict need for second-line therapies or colectomy (see Table 1).

In 1955, Truelove and Witts developed the first sets of criteria to evaluate ASUC and guide intervention. These include a combination of stool frequency (≥6 per day) along with a marker of severity including (heart rate >90 bpm, temperature >37.8 °C, Hb <105 or CRP >30 mg/L) [7]. The risk of colectomy is directly linked to the number of variables present at key timed evaluation point (i.e., Day 3, 5), or 50% colectomy risk when ≥3 criteria are present.

Multiple reassessment tools were developed over the years. The most commonly used is the Oxford Criteria [8] which was developed to re-assess patients treated with first-line therapy at Day 3 of IV corticosteroids treatment. The latter assess stool frequency and CRP (>8 stools/24 h or 3–8 stools/24 h and CRP >45) to determine risk of inpatient colectomy. On Day 7, >3 stools per day or visible blood had a 40% rate of colectomy in ensuing months. The Ho index or Edinburgh risk score is another risk prediction tool that has recently been validated to use on initial presentation of ASUC. It assesses the mean stool frequency over the first 3 days of admission, presence of colonic dilatation (>5.5 cm) and hypoalbuminaemia on admission (<30 g/L). A score > 4 on Day 3 of IV corticosteroids therapy predicts first line treatment failure (sensitivity 85%, specificity 75%) and need for second-line therapy in 66% and colectomy in 33% [9,12]. Seo Index [10] includes several variables including stool frequency, haematochezia, erythrocyte sedimentation rate (ESR), haemoglobin (Hb) and albumin. These variables make up an “activity index” (AI) calculated as such: AI = 60 × bloody stools + 13 × bowel movements/day + 0.5 × ESR − 4 × Hb(g/dL) − 15 × Albumin + 200. Index values reflect disease severity (mild < 150, Moderate 150–220, severe > 220 points). It is calculated on Day 7 of IV corticosteroid and has a positive predictive value of 52% and a negative predictive value of 97% for colectomy when >180 points is reached. Finally, the Swedish index, also known as the fulminant colitis index, uses a formula including stool frequency and CRP (stool frequency/day + 0.14 × CRP (mg/L)); this index has a positive predictive value of 72% for colectomy at a cut-off score of >8 on the third day of corticosteroid therapy [11].

To better assess the mucosa (i.e., presence of deep ulcerations) and rule out other aetiologies (i.e., cytomegalovirus—CMV), an unprepared flexible sigmoidoscopy and biopsy are usually performed within 24 h of a presentation [13,14]. A colonoscopy is usually avoided due to increased risk of perforation [15]. Clostridium difficile should also be excluded in all patients [16]. Ulcerative Colitis Endoscopic Index of Severity (UCEIS) and Mayo endoscopic score (MES) are both used to grade the severity of inflammation in UC. A recent 2018 study showed UCEIS score outperforms MES in ASUC. When UCEIS score ≥7, 80% of patients will eventually need a colectomy as inpatient or during follow-up [17]. In addition, a UK study [18] of 89 patients with ASUC showed a UCEIS score ≥5 more often required rescue therapy compared to those with a UCEIS score <4 (50% versus 27%, *p* = 0.037); similarly, patients with a UCEIS ≥5 had higher rate of colectomy (33% vs. 9%, *p* = 0.012).

## 3. Medical Management of ASUC

Medical management usually precedes any surgical intervention, with the aim to induce remission of ASUC. IV corticosteroids, CsA, and IFX are the main therapeutic options in use today. However, old and new molecules are being considered in the treatment of ASUC, including tacrolimus (TAC), tofacitinib (TOF), and vedolizumab (VDZ). Selected studies are included in Table 2.

### 3.1. First Line Medical Therapy: Intravenous Corticosteroids

The landmark trial by Truelove and Witts [7] support the use of corticosteroids as first line medical therapy in ASUC. They randomized 213 patients to receive 100 mg of cortisone daily vs. placebo for a period of 6 weeks. Higher rates of remission were achieved in the treatment group (41% vs. 16%, *p* < 0.001), along with lower mortality rates (24% vs. 7%, *p* = 0.02) as well as improved endoscopic appearance. Subsequently, a 1974 follow-up study by Truelove and Jewell assessed and evaluated an IV steroids regime in 49 ASUC patients and found 36 (or 73%) were in complete remission at Day 5 [19].

A 2007 systematic review and meta-regression [1] of 32 cohort studies and randomized controlled trials between 1974 and 2006, assessing outcomes of corticosteroids in 2000 patients with ASUC, reported a pooled response rate to steroids of 67%. Moreover, 27% of these patients required a colectomy in the short term (range 5–60 days, or during admission). Colectomy rate was slightly higher in studies where the need for colectomy was evaluated within 2 weeks (32% (95% CI, 28%–36%)) as compared with those in which it was evaluated after 2 weeks of IV corticosteroids (28% (95% CI, 26%–30%)), but this did not reach significance (*P* = 0.13, odds ratio, 1.2 (95% CI, 0.95–1.5)).

The available guidelines reflect this evidence and acknowledge treatment with IV corticosteroids as first line therapy. There is some variability with the type of IV steroids used, e.g., methylprednisolone versus hydrocortisone. There is no additional benefit for higher doses than methylprednisolone 60 mg/day beyond 7–10 days of therapy as it may actually increase complications risk. Hydrocortisone (100 mg every 6 h) has been associated with higher rates of hypokalaemia [36].

### 3.2. Second Line Medical Therapy or “Rescue Therapies”

When IV steroids have failed to improve symptoms by Day 5, one must consider initiating second line therapies for ASUC. These include calcineurin inhibitors (CsA and TAC) and IFX. However, tight monitoring and frequent reassessment of patients are key. Care delivered by primary treating gastroenterologist may decrease adverse outcomes and has been shown to prevent deaths [5]. Delaying surgery in severe patients with suboptimal response will increase the risk of surgical complications and death [37].

#### 3.2.1. Cyclosporine

Cyclosporine (CsA) is a calcineurin inhibitor which has historically been used as a long-term bridge therapy between IV steroids and azathioprine (AZA) in ASUC, or as alternate treatment in patients with contraindication to steroids. A 2003 Belgian study [22] demonstrated a response rate of >80% with doses of 2 mg/kg/day of CsA. Previously used higher dose of 4 mg/kg/day did not have a treatment benefit but was shown to have higher rates of adverse events. Usually, IV CsA is then stepped-down to oral CsA (5 mg/kg) for outpatient management for a period of 3 months as steroids are weaned and/or AZA or mercaptopurine takes effect. Despite its rapid onset of action and efficacy, CsA does not tend to be a preferred second-line therapy in the modern era of biologics due to its onerous frequent drug levels monitoring and adverse side effect profile. There are risks of nephrotoxicity, seizures (associated with low serum cholesterol), electrolyte abnormalities, hypertension, paraesthesia, gingival swelling and serious opportunistic infections [38].

#### 3.2.2. Tacrolimus

Tacrolimus (TAC) is also an inhibitor of calcineurin, ultimately causing a decrease in production of IL-2 and T-lymphocytes. A randomised trial comparing treatment with TAC versus placebo in 62 steroid-refractory moderate-to-severe UC patients showed 50% response rates (vs. 13% in placebo, *p* = 0.003) and 44% mucosal healing rates (vs. 13%, *p* = 0.012) following 2 weeks of therapy with TAC [24]. The latter study reported no statistically significant difference in adverse events between TAC and placebo group. Baumgart et al [39]. reported 78% rate of remission in a group of 40 steroid-resistant UC patients. A systematic review and meta-analysis by Komaki et al [40] in 2016 evaluated two randomised controlled trials (RCT) and 23 observational studies comparing TAC to placebo as rescue therapy in ASUC. Clinical response at 2 weeks was higher with TAC compared with placebo (RR 4.61 (95% CI 2.09–10.17, *p* = 0.00015)) in RCTs. Observational studies reported rates of clinical response at 1 and 3 months as 0.73 and 0.76, and colectomy rates at 3 and 12 months as 0.84 and 0.69 respectively. Liu et al [41] performed a pooled analysis of 6 studies using TAC and IFX in ASUC. The pooled clinical remission rate in TAC-treated patients vs. IFX-treated patients was 52.4% and 48.8% respectively (pooled OR 0.92 (95%CI 0.63–1.34, *p* = 0.66)); the pooled colectomy rate was 10.1% vs. 12.4% (pooled OR 0.86 (95% CI 0.39–1.93, *p* = 0.72)); the pooled adverse events was 44% vs. 19.5% in IFX treated patients (Pooled OR 2.16 (95% CI 1.25–3.76, *p* = 0.006)).

As such, TAC could be considered as an alternative to AZA following a CsA therapy, to assist in maintaining disease control when using biological agent with delayed onset of action (i.e., VDZ) in steroid-refractory UC patients.

#### 3.2.3. Infliximab

The new era of biological molecules has changed the landscape of medical therapies in inflammatory bowel diseases offering satisfactory outcomes with reduced side effect profile. IFX is a chimeric monoclonal antibody against human tumour necrosis factor alpha (Anti-TNF) which has been extensively studied in ASUC. Jarnerot et al [28] completed the first randomized double-blind trial of IFX versus placebo in severe to moderately-severe UC not responding to conventional therapy. Out of 45 patients, 24 were randomised to the IFX treatment arm (single dose 5 mg/kg), and seven out of these 24 patients (29%) had a colectomy versus 14/21 (67%; *p* = 0.017) of patients receiving placebo, within 3 months following randomisation. No serious side effects were reported. Of note, patients in the study had received only a single dose of 5 mg/kg IFX and patients benefited only if they were included based on the Seo criteria (0%), but not based on the Swedish index (47%). Most of the colectomies occurred within 2 months. Long term follow-up (after 3 years) of the same cohort has shown 50% (12/24) of patients treated with IFX had a colectomy vs. 76% (16/21) of placebo. (*p* = 0.012) [29]. This is however not the dosing regimen we usually use in the everyday practice today.

Dosing regimen for IFX in UC is are based on landmark trials ACT 1 and 2 [42] which showed IFX to be more effective than placebo in achieving clinical response in moderate-to-severe outpatients with UC. There was no efficacy advantage to treat with higher 10 mg/kg dose in these original trials, however, these studies specifically excluded hospitalized patients, including ASUC. Meanwhile, it has been postulated the inflammatory burden in severe colitis cause intestinal protein loss and a subsequent state of hypoalbuminaemia, consequently increasing IFX drug clearance [43]. Patient with more severe disease at baseline have been shown to have higher faecal loss of IFX, contributing to primary non-response in UC [44] and possibly to an increased risk of immunogenicity [45]. Further evaluation of IFX dosing regime in ASUC has been retrospectively evaluated, comparing standard dosing (5 mg/kg infusions at week 0,2,6) versus “accelerated” dosing. The latter has variable definitions across studies (5–10 mg/kg infusions over a shorter interval usually <4 weeks) causing significant heterogeneity in the data. Australian data from a multicentre study found no difference in colectomy rate at 3 and 12 months when comparing and 9 ASUC patient treated with accelerated IFX regime (3 × 5 mg/kg infusions within 20 days) vs. 26 patients treated with standard IFX regime (3 × 5 mg/kg infusion at week 0,2,6) [46]. Meanwhile, a study by Gibson et al. retrospectively reviewed 50 steroid-refractory ASUC patients and observed lower rates of early colectomy (3 months) with the introduction of an accelerated (defined as 3 doses 5 mg/kg within 24 days) IFX regime vs. standard dosing (40% vs. 6.7%, *p* = 0.039); there were no difference in colectomy rates at 1 year however [47]. In a propensity score-matched cohort study, Shah et al [48] found initial dosing with higher dose (10 mg/kg) IFX compared to standard dosing is associated with a lower likelihood of needing accelerated IFX induction dosing; the latter was associated with a significantly higher 30-day colectomy rate compared to non-accelerated dosing (*p* = 0.001). A 2019 meta-analysis [49] evaluating 213 patients (132 standard IFX dosing vs. 81 accelerated IFX dosing) with similar baseline characteristics and found no differences in colectomy rates up to 24 months. However, a sub-analysis among those receiving accelerated dosing regime, upfront high dose IFX (10 mg/kg) reduced the short and long-term risk of colectomy up to 2 years, compared to 5 mg/kg dosing. The recently published McGill [50] experience of 72 ASUC patients further supports that higher dose IFX (10 mg/kg) induction vs. standard, does not improve 3 months colectomy rates (14% vs. 5%, *p* = 0.205).

A 2019 Systematic review and meta-analysis [51] performed a sub-analysis of all data available to date which showed dose-intensified induction was not significantly different compared to standard induction, despite being used in a subset of patients with more severe disease. Their analysis highlights the variability in IFX salvage therapy and the need for further randomized prospective studies to guide optimal IFX dosing and clinical strategies in hospitalized acute severe UC such as the Australian ongoing PREDICT-UC (NCT02770040) study.

Biosimilars are replacing IFX worldwide. Two studies evaluating the biosimilar CT-P13 in ASUC reported no differences in outcomes compared to the original molecule [52,53]. Other biologics such as adalimumab and golimumab are used for treatment of moderate-to-severe UC; however, there is no data for their use in ASUC specifically.

#### 3.2.4. Cyclosporine Versus Infliximab

Selection of rescue therapy is based on several factors including efficacy, safety profile and patient and/or provider preference and experience of use. CsA has a rapid onset of action, a shorter half-life (7 h vs. 9 days for IFX) and consequently can be used to transition to another agent, including a biologic, thereafter. IFX is usually thought to have a better side effect profile and ease of use and monitoring.

In terms of comparing efficacy between CsA and IFX, two randomized trials assessed these rescue therapies in ASUC: CYSIF [3] and CONSTRUCT [33]. The CYSIF study by the GETAID group randomized 115 hospitalized steroid-refractory UC patients who failed 5 days of IV steroids. The study concluded a single 5 mg/kg dose was not inferior to 7 days of CsA in the short-term. Furthermore, there were no difference in overall colectomy rate between IFX and CsA (21% in IFX vs. 17% in CsA), nor time to colectomy (*p* = 0.6). Interestingly, side effects profiles of the two molecules were largely similar. Similar findings occurred in the larger CONSTRUCT trial (*n* = 270 patients) from the UK where 9 patients treated with CsA required IFX within a year, versus on IFX needed CsA. Overall the CONSTRUCT trial showed no significant difference between CsA and IFX for clinical efficacy or colectomy rates or adverse events. In contrast to the RCT data, a systematic review of 13 observational studies [4] suggest IFX was associated with a higher rate of treatment response and a lower 12 month colectomy rate compared with CsA. There were however no significant differences in adverse drug-related adverse events, post-operative complications or mortality.

Overall this data supports the use of either agents as second-line therapies in ASUC, but emphasis should once again be placed careful patient selection and assessment, patient preference, and provider experience.

### 3.3. “Third Line” Medical Therapy or Sequential Therapy

Third line therapy or sequential therapy should preferably be considered in expert IBD centres on a case-by-case basis. Significant adverse events and death have previously been reported with sequential therapy from CsA to IFX [54].

Long-term data on outcome of ASUC patients treated with CsA or IFX show colectomy-free survival was independent from initial treatment [31]. Of note however, 46% of patients initially treated with CsA needed IFX at 1 year, and 57% at 5 years; in contrast, only four patients initially treated with IFX were switched to CsA. Several studies evaluated sequential therapy from CsA to IFX with no sinister outcomes, in IBD as well as psoriasis [55]. Practically speaking, IFX is preferred as second-line therapy. Weisshof et al. assessed 40 steroid and IFX-refractory ASUC patients receiving sequential therapy with CsA; 60% achieved clinical remission within 2 weeks, and 42% had colectomy-free survival at 1 year, with no increased in adverse events, which suggest CsA therapy following rescue therapy failure with IFX can be effective and safe in ASUC [56]. A recent publication in a mixed cohort of steroid and/or anti-TNF refractory UC +/− ASUC (*n* = 39) has shown safety and efficacy of bridging patients from third line CsA to VDZ, with 68% colectomy-free rate at 12 months follow-up [57].

A systematic review of ten studies, or 314 participants, showed sequential treatment with combinations of steroids, CsA or TAC, and IFX, led to ASUC patients achieving a response in 62% of cases and remission in 39%; colectomy rates were 28% at 3 months, but were as high as 42% at 12 months. Adverse events were encountered by 23% of patients, including serious infections in 7% and mortality in 1% [58]. This study highlights that the quality of the evidence available overall is low and consequently is unable to draw definite conclusion on appropriate sequence of therapies; we may be able to delay rather than prevent colectomies, but it has to be clearly balanced with the higher risks of adverse outcomes.

Current consensus statements from Australia [59] and Canada [2], do not support the use of sequential therapies generally due to its increased risks of adverse events and infections, and delay of surgical intervention.

### 3.4. Other Medical Therapeutic Options

#### 3.4.1. Tofacitinib

Tofacitinib (TOF) is a small molecule that blocks Janus kinase (JAK) 1, 2, and 3. This inhibition leads to a decrease in pro-inflammatory cytokines production. The OCTAVE trials (Octave 1, 2, and Sustain) proved the efficacy of TOF for induction and maintenance of moderate-to-severe UC whom have failed previous therapy with anti-TNF [60]. Moreover, a post-hoc analysis from the Phase III trials demonstrated efficacy of TOF over placebo in inducing remission over a three-day period [35].

Very limited data exists in TOF use in ASUC; however, Berinstein et al [34] recently published a case series of four patients with ASUC treated with TOF where three out of four patients were able to achieve clinical remission and 50% were able to avoid colectomy.

#### 3.4.2. Vedolizumab

Vedolizumab (VDZ) is a selective antibody against α4β7-integrin, which targets leukocyte trafficking in the gastrointestinal tract. Efficacy of VDZ in induction and maintenance of moderate-to-severe ulcerative colitis was demonstrated in the Phase III randomized, placebo-controlled trials GEMINI 1 [61]. At week 52, 42% of patients achieved clinical remission on 8-weekly VDZ infusions. Moreover, VDZ has a safer adverse event profile than other molecules due to its selective inhibition; this makes it a molecule of choice in sequential therapy. However, there is no data evaluating VDZ in the ASUC-specific cohort.

### 3.5. Emerging Therapies in Development

There is currently no published data on emerging therapies in the specific cohort of hospitalized acute severe UC. However, several new molecules are under investigation in moderate-to-severe UC, assessing different molecular targets including JAK1 (i.e., Upadacitinib (UPA)), sphingosine 1 phoshate (S1P) receptor modulator (i.e., Etrasimod, Ozanimod), anti-integrin monoclonal antibodies (i.e., Etrolizumab, AJM 300), anti-Interleukin(IL)-23 (i.e., Mirikizumab). Most of the molecules have completed Phase II trials and are entering into Phase III recruitment, with the exception of ustekinumab (UST) which has completed Phase III trials and is pending mainstream approval imminently. The recent Phase III UNIFI trials [62] showed that UST, an antagonist of the p40 subunit of IL 12 and IL-23, was more effective than placebo at inducing ((UST 15% vs. Placebo 5%), *p* < 0.001) and maintaining (8-weekly UST 44% vs. placebo 24%, *p* < 0.001) remission in a population of moderate-to-severe UC patients who are refractory to anti-TNF, VDZ or conventional non-biologic therapy. A milder side effect profile, along with a low immunogenicity rate and subcutaneous dosing makes UST an attractive therapy for UC patients.

Alteration of the intestinal microbiota with fecal microbiota transplant (FMT) has been evaluated as a therapeutic option, due to emerging evidence supporting the dysbiosis theory underlying IBD. However, the data available on FMT in UC is quite heterogeneous, as a consequence of the different delivery methods used (colonoscopy vs. nasogastric tube vs. enema), dosing interval regimens, and stool sampling characteristics (pooled vs. single donor vs. “super-donor”). Moreover, the patient population included in trials tend to have relatively small study sample sizes, and include mild-to-moderate UC on maintenance treatment with mainstream therapies (5-ASA, AZA, Methotrexate, Anti-TNF, VDZ, +/− Prednisone). A systematic review and meta-analysis [63] of the 4 key RCTs on FMT in UC demonstrated an overall pooled rate of clinical remission (42% FMT-treated vs. 23% control, NNT = 5 (95% CI 3–17)) and endoscopic remission (26% FMT treated vs. 10% control) which favoured FMT treated patients over controls. There was no significant difference in adverse events between cohorts. Long-term safety and efficacy data are limited and needs to be evaluated further. There are several clinical trials underway on an international scale to assess FMT in IBD in general.

Given the modest efficacy rates of current biologic therapies and novel small molecules, the future of IBD management is likely to entail combination therapy using different molecules and/or microbiota-altering methods concurrently.

## 4. Surgical Management

Restorative proctocolectomy with an ileal pouch anal anastomosis (IPAA) is the gold standard procedure for ulcerative colitis patient since its introduction in 1978 [64]. IPAA is usually performed in two stages (total proctocolectomy with IPAA and diverting ileostomy, followed by ileostomy closure), or three stages (first subtotal colectomy, then completion proctectomy with IPAA and diverting ileostomy, then ileostomy closure). A recent single-centre study comparing outcomes of 212 UC patients (from year 2000–2015) undergoing 2-stage (*n* = 157) compared to 3-stage (*n* = 55) IPAA have comparable outcomes (complications, anastomosis leak, pouchitis, number of bowel movements or sexual satisfaction) and quality of life 6 months following ileostomy reversal [65]. A systematic review of 33 studies encompassing 4790 patients evaluated health-related quality of life (HRQoL) and health status (HS) in UC patients after restorative proctocolectomy with IPAA, and showed that 12 months post-operatively HRQoL and HS return to general population level [66]. Good quality of life and functional outcomes have been showed in patients with IPAA in a recent Swedish study [67]. In young female of reproductive age, it is commonly advised to maintain the ileostomy and complete their family prior to pelvic surgery as it is associated with reduced fertility and increased use of in vitro fertilisation (IVF) by a factor of three [68,69].

Surgery may be indicated early in patients with ASUC, and delaying surgery can increase the risk of surgical complications and mortality [37]. In ASUC, the primary aim is to save the patient, not necessarily the colon. ASUC patients are best managed by a multidisciplinary team, including a gastroenterologist, colorectal surgeon, dietician, pharmacist, specialised IBD nurse, and stoma nurse. It is of utmost importance that colorectal surgeons be involved early in the management of ASUC patients and participate in the decision-making on sequential salvage therapy versus colectomy [59,70]. Meanwhile, whilst UC inpatients outcomes and mortality are improved when under the care of a gastroenterologist, there is no decrease in the risk of colectomy [5].

A recent study by Moore et al [71] evaluated the predictive value of the Oxford criteria in 80 ASUC patients who received second line therapy. Patients fulfilling the Oxford criteria had a higher risk of colectomy during hospital admission (12/33 (36%) vs. 4/41 of those who did not meet the criteria (10%), *p* = 0.009). Nevertheless, it is worth noting that the risk of acute colectomy has been significantly reduced compared to the pre-biological era of 85%. (according to Oxford criteria). However, colectomy in the context of ASUC carries a higher postoperative mortality risk up to 5.3% [6].

## 5. Conclusions

ASUC is a medical emergency, and the goal of medical therapy is to reduce active inflammatory process and avoid colectomy. Nevertheless, providers need to cautiously balance the risks of prolonged suboptimal (sequential) medical therapy compared to the morbidity and mortality associated with an emergency, often delayed, colectomy. Sequential therapy may increase adverse events and peri-operative complications and should be reserved for use in expert IBD centres. Outcomes are superior if the primary treating physician is a gastroenterologist, and when IBD specialists and colorectal surgeons are involved early in the medical management emphasising the need for a multidisciplinary approach to care. The primary aim should be to reduce patient mortality over saving the colon. New therapeutic options are being developed and assessed, and will encourage a more personalized treatment approach in UC. However, further evaluation of these therapies in ASUC are needed. 

## Figures and Tables

**Table 1:** Acute Severe Ulcerative Colitis Prognostic Scores.

Prognostic Score	Variables Considered	Predicted Colectomy Rates
Truelove and Witts [7] (Use Day 1)	Stool frequency Haematochezia Heart rate Temperature Haemoglobin CRP	50% risk of colectomy when 3+ variables are present
Ho Index [9] (Use Day 3)	Stool frequency Colonic dilatation Hypoalbuminaemia	Score ≥4 predicts need for second line therapy in 66% and colectomy in 33%
Oxford Score [8] (Use Day 3)	Stool frequency > 8/day or Stool frequency 3–8/day and CRP > 45	PPV 85% (pre-biologic era)
Seo Index [10] (Use Day 3)	Stool frequency Haematochezia Haemoglobin Albumin ESR	PPV 52% NPV 97% when >180 points
Swedish index [11] (Fulminant Colitis Index) (Use Day 3)	Stool frequency CRP	PPV 72%

CRP = C-Reactive Protein; ESR = Erythrocyte Sedimentation Rate; NPV = Negative Predictive Value; PPV = Positive Predictive Value.

**Table 2:** List of selected studies evaluating different biologics and small molecules in adult patients with ASUC.

Author (year)	Study Type	Study Population	Outcome
**Corticosteroids**			
Truelove and Witt (1955) [7]	RCT Oral cortisone vs placebo	Chronic ulcerative pancolitis	Clinical response 42% vs. 13%; Mortality rate 7% vs. 24%
Truelove and Jewel (1974) [19]	Uncontrolled trial (IV steroids)	Acute severe UC (*n* = 49)	73% clinical remission; 27% colectomy rate
**Cyclosporine**			
Lichtiger (1994) [20]	RCT CsA (4 mg/kg/day) vs. Placebo	Steroid-refractory UC 11 CsA vs. 9 placebo	82% response with CsA vs. 0% response in placebo
D’Haens (2001) [21]	RCT CsA (4 mg/kg/day) vs. steroids	ASUC 15 CsA vs. 15 steroids	At Day 8, clinical response in 64% CsA and 58% steroids; At 12 mths, 78% CsA remained in remission vs. 37% steroids-treated group
Van Assche (2003) [22]	RCT CsA (2 mg/kg/day) vs. CsA (4 mg/kg/day)	ASUC 35 low-dose vs. 38 high-dose	82% vs. 83% response in the 2 mg/kg/day vs. 4 mg/kg/day group; 14 days Colectomy rate 8.6% vs. 13.1% (low vs. high dose)
**Tacrolimus**			
Ogata (2006) [23]	RCT TAC vs. Placebo (TAC serum concentrations 10–15 ng/mL)	Steroid-resistant UC 19 TAC vs. 20 placebo	13/19 clinical response with TAC vs. 2/20 placebo; 0% clinical remission at 2 weeks in both groups
Ogata (2012) [24]	RCT TAC vs. placebo	Steroid-refractory mod-severe UC 32 TAC vs. 30 placebo	50% response rate vs. 13% in placebo (*p* = 0.003); 44% mucosal healing rates (vs. 13% placebo, *p* = 0.012) at 2 weeks.
**Tacrolimus vs. Infliximab**			
Yamagami (2017) [25]	RCT TAC vs. IFX	Moderate-severe UC 64 TAC vs. 58 IFX	Clinical remission 50% TAC vs. 38% IFX
**Infliximab**			
Sands (2001) [26]	Pilot study/RCT IFX vs. placebo (1× IFX 5 mg/kg)	Severe steroid-refractory UC 8 IFX vs. 3 control	3 months colectomy rate: 50% in IFX vs. 100% in Control (*p* > 0.05)
Ochsenkuhn (2004) [27]	Randomised pilot study IFX vs. prednisolone (3× IFX 5 mg/kg)	Acute severe UC (non refractory to steroids) 6 IFX vs. 7 prednisolone	At 3 weeks follow up, colectomy rate 0% in IFX and control group (*p* = NS)
Jarnerot (2005) [28] Gustavsson (2010) [29]	RCT IFX vs. placebo IFX single infusion (4–5 mg/kg)	Moderate-severe UC steroid refractory 24 IFX vs. 21 control	3 months colectomy rate: 29% in IFX vs. 67% in control (*p* < 0.05); 3 years colectomy rate: 50% in IFX group vs. 76% control
**Cyclosporine vs. Infliximab**			
Bossa (2009) [30]	RCT IFX vs. CsA (3× IFX 5 mg/kg)	Steroid-refractory ASUC 14 IFX vs. 7 CsA	1 month colectomy rate: 43% vs. 43% (*p* = NS)
Laharie (2012) [3] Laharie (2018) [31] CySIF	RCT IFX vs. CsA (3× IFX 5 mg/kg)	Steroid-refractory ASUC 57 IFX vs. 58 CsA	3 months colectomy rate: 21% IFX vs. 17% CsA (*p* = NS); 5 years colectomy rate: 35% IFX vs. 39% CsA (** *note: 46% of CsA-treated pts switched to IFX by 1 yr to maintain remission*)
Croft (2013) [32]	Prospective cohort IFX vs. CsA (1× IFX 5 mg/kg)	Steroid-refractory ASUC 38 IFX vs. 45 CsA	3 months colectomy rate: 24% vs. 47% (*p* = 0.04); 1 year colectomy rate: 35% vs. 58% (*p* = 0.04)
Williams (2016) [33] CONSTRUCT	RCT IFX vs. CsA (3× IFX 5 mg/kg)	Steroid-refractory ASUC 135 IFX vs. 135 control	2 years colectomy rate: 41% IFX vs. 48% CsA (*p* = NS)
**Tofacitinib**			
Berinstein (2019) [34]	Case reports	4 steroid or IFX-refractory UC patients	75% clinical remission; 50% colectomy rate
Hanauer (2019) [35]	Post-hoc analysis of OCTAVE 1 and 2 trials	Moderate-severe UC steroids, AZA and/or IFX refractory (TOF vs. placebo)	By Day 3, improved Mayo stool frequency and rectal bleeding subscores. Associated with PPV of response at week 8.

ASUC = Acute Severe Ulcerative Colitis; AZA = Azathioprine; CsA = Cyclosporine; IFX = Infliximab; IV = Intravenous; NS = Not Significant; *p* = *p*-value; RCT = Randomised Controlled Trial; TAC = Tacrolimus; TOF = Tofacitinib; UC = Ulcerative Colitis.
